# Cerebral Pathology

**Published:** 1855-01-01

**Authors:** 


### CEREBRAL PATHOLOGY.

Dr. Semple read a paper on the subject of *The Pathology and Diagnosis of Cerebral Diseases*, before the Medical Society of London, Nov. 14, 1853. He commenced by observing, that the present paper might be considered as a continuation of a former communication to the Society on the same subject. He did not intend to present an elaborate essay on cerebral pathology, nor to avail himself of the opinions of authors who had written upon the subject, but merely to present the results of his own experience and observations; particularly selecting those points which were the most obscure, or on which a difference of opinion still existed. It was often a matter almost of impossibility to connect the symptoms of cerebral disease observed during life with the appearances found after death; but, as the term "pathology" included the morbid phenomena of the living structure, as well as the lesions traced after death by the scalpel, it was necessary to consider them in their relations to each other, however obscure the relationship might appear. In some cases, the relation of cause and effect was sufficiently manifest; and Dr. Semple drew attention, in the first place, to diseases of the osseous structure as causes of cerebral disorders. In one case which had come under his notice, and which he had observed for about ten years, the patient had been subject to continual attacks of epilepsy, which had resisted all treatment, and which had at last proved fatal. On a *post-mortem* examination, it was found that the internal table of the skull was thickened, and all the prominent bony processes in the interior of the cranium were much increased in thickness and asperity, as the cristi galli, the clinoid processes, the ridges on the petrous portion of the temporal bone, and on the occipital bone. In another case, he attended a patient who suffered from constant pain and heaviness at the anterior and inferior part of the forehead, and who had occasionally had epileptic fits. These inconveniences became so troublesome by their long continuance, that the sufferer was obliged to abandon his business, and life became a burden to him. Acting upon the supposition that this was a case of thickening of the internal table of the skull, mercury was given to salivation, and the iodide of potassium was administered in large and repeated doses for a very long period, and under this treatment the patient eventually recovered, and is now in active occupation. In a third case, a man presented a manifest thickening of the root of the nasal bones; and notwithstanding the vigorous employment of the means pursued in the last case, he became comatose and died; and on a *post-mortem* examination, there was found to be thickening of the bones in the region indicated, and an abscess in the brain corresponding in situation to the osseous lesion. Another circumstance which Dr. Semple had occasionally observed in cerebral diseases, was a want of symmetry in the two sides of the cranium; and although he was not yet prepared to prove that this was a common cause of cerebral disease, yet he thought it worthy of attention, and believed that other cases might be found in which similar deviations from symmetry might prove to be associated with disordered cerebral manifestations. The morbid anatomy of the cerebral membranes is a subject involved in great obscurity, because the most violent symptoms during life often leave very few traces observable after death; and, on the other hand, appearances usually described as morbid are sometimes wholly unconnected with any cerebral symptoms during life. In order to arrive at correct views on this subject, however negative the results might be, the author was in the habit of examining the brains of all cases, whenever it was practicable, whether there had been symptoms of cerebral disorder during life or not, and the conclusion at which he had arrived by these investigations was, that much error and misapprehension existed among authors on this branch of pathology. Such terms as "more or less opacity of the arachnoid," "effusions on its surface," "effusions beneath its surface," and other similar vague expressions, were by all means to be deprecated, because it was doubtful whether these appearances were really morbid at all in many instances, inasmuch as they are often found in cases where no cerebral disease has existed. The disease called acute hydrocephalus in children, was a well-marked instance of meningeal inflammation; and the results of such disease were apparent in the effusion of coagulable lymph between the opposed layers of the arachnoid, and of copious serous deposit upon the surface and in the cavities of the brain; but in adults, the effusion of coagulable lymph was comparatively rare, and the only appearances observed were vascularity of the membranes and effusion of lymph beneath the arachnoid. Dr. Semple inclined to the view of Rokitansky, that inflammation of the pia mater is the usual form of meningeal disease, the apparent vascularity of the arachnoid membrane being due to the injection of the vessels of the pia mater seen through the transparent serous structure lying above it. Four cases were then related, in two of which the author considered that meningeal inflammation undoubtedly existed; and yet after death nothing was found beyond a slight effusion of lymph beneath the arachnoid, and in the two others no cerebral disease had existed during life, and yet the *post-mortem* examinations revealed thickening and vascularity of the arachnoid, accompanied by copious effusion. The diagnosis of cerebral diseases is a point of great difficulty, but of immense importance, and upon a correct judgment in this particular the life or death of a patient often depends. Insensibility and coma depend on various causes,—such as hysteria, poisoning, drunkenness, apoplexy; and all these conditions require very different and often opposite treatment. Again, delirium is produced by opposite states of the brain—by congestion and inflammation, on the one hand; by exhaustion of nervous energy on the other; and some well-marked cerebral disorders are produced by diseases in remote organs, and without any disease of the brain whatever. Two cases of what might be called pseudo-cephalic disease were then related, in one of which the most violent and long-continued convulsions were caused by the eccentric irritation of teething and intestinal derangement; in the other, an infant a fortnight old, the convulsions were apparently due to previous intra-uterine influences, and to the weak and nervous condition of the mother. In such cases, it was exceedingly important that a correct diagnosis should be made; for, although convulsions in infants were always dangerous, and a prognosis must be given with great caution, yet the gravity of the issue was most materially influenced by the nature of the existing disease. The convulsions springing from cerebral inflammation must be promptly treated by leeching, calomel, purgatives, cold to the head, warmth to the extremities; but all these remedies are utterly improper in those forms of convulsive attacks which originate from teething, intestinal derangement, renal obstructions, and other forms of eccentric disease.

The chief point in the discussion was the degree of difficulty attending the diagnosis of cerebral diseases.

Dr. Fuller was of opinion that in true meningitis there is always great congestion, with effusion of lymph, pus, or serum; and that in the absence of these products, we could not infer the existence of the disease from the symptoms. In poisoned blood from disease of the kidney, or from the presence of a narcotic poison, as belladonna, as also in certain rheumatic affections, he had observed great cerebral excitement, with injection of the conjunctiva—that is, all the mere symptoms of meningitis—but these were not true cases of inflammation. Moreover, the opacity of the arachnoid, which arises from an effusion underneath the membrane, is not an evidence of inflammation. He then referred to the probable cause of the greater violence of the cerebral irritation in meningitis than in cerebritis, and believed it to be the rapidity with which the various disturbances take place in meningitis. He had observed cases in which very great injury had been done to the substance of the brain, without any violent symptoms having been induced, and this he attributed to the slowness with which the injury had proceeded. Thus, in the case of a medical friend, whe for many years had suffered only from symptoms resembling tic, and from slight epileptic attacks, he had found a pint of serum effused within the skull, with the circulation through the right vertebral artery impeded by the pressure of a scrofulous tumour, and the left vertebral artery nearly obliterated. He agreed with the author in his remarks upon disease of the calvarium, and instanced the case of a young lady who had suffered for many years from intense headache and epileptic fits, and who could not bear any jolting exercise, in whom a spiculum of bone, two inches in length, was found projecting into the brain.

Dr. Theophilus Thompson considered that thickening of the calvarium was not a likely cause of meningitis, for he had observed that thick-skulled people were dull, and not prone to inflammation; but if it were a cause, he did not think that iodide of potassium would remove it. He believed that affections of the dura mater are among the most obscure of cerebral diseases, while inflammation of the other membranes of the brain is at present well understood. He thought that Mr. Rainey's statement as to the ganglionic character of the arachnoid, would clearly account for the great violence of the symptoms met with in arachnitis, and that it is the locality affected, and not the slowness of the progress, which renders the symptoms of cerebritis less prominent.

Dr. Druitt inquired if the author had been accustomed to connect rheumatism with disease of the skull. He (Dr. Druitt) had given colchicum and calomel with great advantage in such cases, and believed those remedies to be more beneficial than iodide of potassium. He considered that thickening of the skull is rarely, if ever, a primary disease; as also meningitis, when not preceded by external injury. If in any case he should observe great cerebral excitement, with congestion of the eye, he should consider the case to be one of poisoned blood, and not of true idiopathic meningitis.

Dr. Sibson believed it to be impossible to arrange the diagnostic symptoms of each cerebral disease in clearly-defined categories, for he had learned from practice and an analysis of almost all the recorded cases, that every kind of symptom has been found in every variety of cerebral disease. A few cases of epilepsy have thickening of the calvarium, but it is only a small minority; and in general, he believed it to be impossible to guess even at the existence of such an obscure morbid condition. He also considered that inflammations of the arachnoid and of the pia mater could not be separated, just as it is impossible to state that the sub-pleural cellular tissue is not involved in a case of pleuritis. Farther, in such cases, the surface of the brain is always implicated, and it is from this cause alone that the symptoms become more energetic. He regarded the effusion of lymph in a fatal case of meningitis as essential to the disease, and had seen cases in which this effusion had extended greatly, and yet only a slight stupor had indicated the presence of inflammation.—Nov. 16, 1853.

*Simple Ventricular Meningitis.* By W. Hughes Willshire, M.D.—Simple acute meningitis is, under any of its forms, an unfrequent disease in childhood. At this period, the meningeal inflammation is usually of the granular or tuberculous character, or, at any rate, is subservient to the sway of the important diathetic disorder, scrofula. But if simple acute meningitis of the periphery alone, or combined with that of the base or of the ventricles is uncommon, that limited to the lining membrane of the latter is excessively rare. So rare, indeed, is it, that MM. Rilliet and Barthez have been unable to meet with a single case on record. The former, however, has been witness to one, terminating in ventricular effusion, loss of intelligence, idiotcy, and death. The fatal event did not occur till the end of the fourth month, the disease assuming somewhat of a chronic character. The case seems more particularly interesting, as tending to support the views of those who believe chronic internal hydrocephalus to be due to inflammation of the ventricular living membrane. The following instance occurring to ourselves, differs in some important points from M. Rilliet's, and appears more fully to demand the qualification of *acute* to the terms ventricular meningitis.

C. W., a boy five years old, was brought to the infirmary in the month of January. His parents live near the institution. The child was said to have been ill for more than a week, and to have been an out-patient at the Charing-Cross Hospital. The prescription-paper of the latter showed that antimonials and salines had been given. The patient was very thin, pale, and weak, lying in his mother's lap, scarcely able to speak, though complaining somewhat of his head. There was thirst, loss of appetite, coated and rather dry tongue, but no costiveness. On a review of the whole symptoms, and being impressed by the recollection of an epidemic then prevalent in the locality, I came to the conclusion that the child was suffering from chronic remittent fever of a low type. Still, I was not quite satisfied with the diagnosis, as there appeared something not easily to be described in the case, different from the patients I was then attending. The epidemic then prevalent absolutely demanded bark and ammonia for its satisfactory treatment, and these agents were here given. Under their employment, a great change for the better appeared to ensue, and progressed for an entire week. I watched the case with much interest, being suspicious about my diagnosis, but at the end of the week I entered in my note-book that I thought my patient would do well, and that "my diagnosis is right." The next day a change appeared. The patient became worse, and complained bitterly of his head. There was no costiveness, rather the reverse, but there was some vomiting. The ammonia and bark were stopped, leeches applied to the temples, and blisters behind the ears, antimonials, salines, &c., given. No relief from any treatment was obtained, the child became still worse, semi-conscious, the pupils were dilated, the head thrown back, and the limbs became slightly stiffened. With slight alternations, these symptoms continued for four days, when the limbs became more relaxed, and every now and then affected with a sort of slow shaking or trembling movement. The remission called the "lightening before death" appeared, then, as was expected, the symptoms became worse, the limbs stiffer, the head thrown back, the hands clenched, and the patient died at the end of the second week since he was first seen at the infirmary.

The case had been very obscure to us; there had been no definite convulsions, no screaming, no "cerebral respiration," though the latter was frequent, no constipation, and but little vomiting. The constant dorsal decubitus, the peculiar opisthotonic symptoms, and the pyrexial prodromi were the more marked positive phenomena—of course the lesion was cerebral, but that was saying little; there was probably effusion, that was not saying much more. It might be the base, or the hemispheres, or the ventricles, which were more particularly involved, or it might be the meninges alone which were affected, or they might be intact, and true tubercle exist of the cerebellum medulla, or brain proper. Farther, the inflammatory element, if present, might be either of the simple or granular character, the fever might be symptomatic of the cerebral mischief, or reactional, or be the primary disorder, and the affection of the nervous centre be secondary to it.

*P. M.*—Skull well ossified, convolutions of brain close-pressed, the membranes intensely congested. No milky effusion along the course of the vessels, no exudation of any kind beneath the arachnoid or upon the hemispheres. No granules along the edges of the latter. Cerebral matter showing numerous red points and striæ on section, but no continuous blush. Ventricles greatly dilated, extending the whole length of the hemispheres, and full of serum. In each posterior cornu floated a thick continuous flock or flake of green-coloured purulent matter. The ventricular lining membrane was thickened and vascular in parts, and rough and broken down elsewhere. No central softening existed. A small quantity of green purulent matter was found at the base. The cerebellum was rather softer than natural. No tubercular deposit existed within the cranium, nor within the thorax.

The absence of the ordinary characters of simple hemispheric acute meningitis, of those of the tuberculous meningeal affection, the slight evidence, comparatively, of lesion at the base, and the very positive signs of the ventricular changes, together with the peculiar symptoms, authorize me, I believe, in considering the above case as one of simple acute ventricular meningitis.

*Abscess of Brain—Disease of the Ear.* By W. Hughes Willshire, M.D.—Disease of the central organs of the nervous system, from more primitive mischief going on in the bony structures adjacent, is of no unfrequent occurrence. This connexion between scrofulous disease of the internal ear and the destruction of the brain, is occasionally illustrated by such an example as the following:—J. F., a boy eleven years of age, and living at Southwark, came under my care in the month of July, 1852. His mother stated that, three weeks before, he went into the country, but returned home ill. He then "had some fits," and soon began to complain of his head. A discharge of matter which had been wont to flow from both ears now stopped, and the pain of the head then became so intense that the boy screamed out from the agony. He then had another fit, and I was now requested to see him. On cross-examination, it appeared that, when two years of age, he had "brain fever," then "inflammation of the ears," and discharge from them, which had troubled him very severely off and on until now. He was of a very scrofulous family. He was quite conscious, lying with his hand placed against the right side of his head, complaining of the pain there. The tongue was coated, the bowels not costive, and there was some fever. Leeches were applied to the temples, blisters behind the ears, and afterwards poultices to the latter organs. Purgatives and full doses of nitre were also given. For four or five days great improvement seemed to follow; so much so, that I began to think the cerebral disturbance had no intimate connexion with the disease of the organs of audition. Suddenly, the patient became worse. I found him moaning from the severity of the pain, and sorely complaining of his head. In answer to my inquiries, I was informed that he had squinted, and "made mouths and strange faces." He constantly cried out for some one to press his head hard; consequently, his mother or some relatives sat at his bed-side for hours together, pressing with their hands upon his head. In this state he remained for two days, not unconscious, but in what might be called rather a stupid condition, and evidently suffering intense pain in the head. Convulsions supervened, and death followed ten days after he began last to complain of the cerebral symptoms. On examination of the body, a large abscess was found in the left cerebral hemisphere, communicating with the ventricle, and filled with green fetid pus. On the other side, its walls approached at one point just close enough to touch the cranial bone connected with the left ear. The bones of the latter were diseased; but not to the extent of causing a communication between the external meatus and the interior of the cranium. Such, however, would apparently soon have occurred, as the bone was becoming carious at the point where, when we were removing the brain, the abscess burst, emitting much of its horribly fetid contents. The rest of the brain generally was anæmic; but the vessels of the meninges were greatly congested. Unfortunately, the exact character of the walls of the abscess was not carefully noted down at the time; but from recollection it is believed it was of a thin cystic description. In this case, it may be asked, how long the abscess had been forming, and why it was the pain so sorely complained of was felt on the right side, whilst the collection of purulent matter existed on the left.—*Ibid.*

*Prognosis and Treatment of Epilepsy.*—The *Union Médicale* for May 17th and 19th, contains an article by Dr. Herpin, of Geneva, on the above subject, of which we now give an abstract.

In the *Union Médicale* for December 1, 2, and 7, 1852, M. Moreau, of Tours, relates nine cases of epilepsy, in which oxide of zinc had failed to arrest the disease, a remedy stated by Dr. Herpin to be of considerable efficacy. Seven of the cases were of the class stated by Dr. Herpin to be most amenable to treatment, and the medicine was administered according to the rules laid down by him in his essay, "Du Prognostic et du Traitement curatif de l'Epilepsie," published last year at Paris. Dr. Herpin points out the causes of M. Moreau's want of success, in the following manner:—

1. The first remarkable point, which may account in a great measure for the different results obtained by M. Moreau and Dr. Herpin, was, that eight of M. Moreau's cases were hospital patients, while Dr. Herpin's were private patients. Dr. Herpin observes, that physicians who have the charge of epileptic wards in hospitals regard the disease as almost always incurable; while those who see the patients at home, as far as can be judged from their writings, form a very different prognosis. Tissot, Odier, De la Rive, and C. Vieusseux, all believe in the curability of a fair proportion of epileptic cases. A principal cause of the difference between the opinions of the two classes of practitioners is, that those in private practice generally see the disease from its commencement, while hospital physicians almost always have to treat severe or obstinate cases.

2. M. Moreau had only male patients; Dr. Herpin had more females than males. From an analysis of his cases, Dr. Herpin arrives at the following results:—

Of twenty-six female epileptic patients, sixteen were cured, six were improved, and four were incurable.

Of twenty-four male epileptic patients, twelve were cured, four were improved, and eight were incurable.

There were thus twice as many incurable cases among males as among females.

3. With regard to age, Dr. Herpin has obtained the following results:—

Of thirty-five patients under 20 years, eighteen were cured, nine improved, and eight were incurable.

Of nine patients aged from 20 to 50, five were cured, one was improved, and three were incurable.

Of six patients aged from 50 to 80, five were cured, and one was incurable.

The period of life from 30 to 50 furnishes a third of incurable cases; while the other two do not together supply a fourth. All M. Moreau's cases were from 19 to 50 years of age, the most unfavourable period.

4. With regard to the previous duration of the disease, Dr. Herpin finds that—

Of twenty-three cases, which had existed less than a year, fifteen were cured, five were improved, and three were incurable.

Of twenty-seven cases from one to twenty years' duration, thirteen were cured, five were improved, and nine were incurable.

While nearly one-half of Dr. Herpin's cases were of less than a year's duration, three of M. Moreau's patients had been ill from fourteen to twenty months, one for two years at least, three for six years, and one for about twenty years; the ninth had recent attacks of vertigo, but had probably had an epileptic attack six months before.

5. With regard to the number of attacks previous to treatment:—

Thirty epileptic patients, who had had less than twelve attacks, furnished only three incurable cases.

Twenty-two patients who had had at least from thirty to a hundred attacks, furnished twelve completely obstinate cases, being at least five times as many as in the preceding category.

Of M. Moreau's nine cases, one, who was seized with vertigo, had perhaps had a fit; one patient had had only four attacks; one had had about fifty; four from seventy to eighty; one more than a hundred, and one more than five hundred. Besides this, one of the patients had, before the commencement of treatment, paralysis, denoting organic lesion of the brain, which was proved by the autopsy; and another had been twice insane. This latter circumstance was met with in one of Dr. Herpin's cases, in whom, though the conditions for treatment were otherwise favourable, the disease remained incurable.

Besides these causes of failure in M. Moreau's cases, Dr Herpin points out that the want of sufficient judgment in the choice of treatment is perhaps a more powerful obstacle. He observes that as long as we are unacquainted with the indications of each remedy for epilepsy, we must begin by giving that which experience has shown to have succeeded in the greatest number of cases; then, in case of failure, we must have recourse in succession to other remedies of efficacy. By employing only one, especially in a number of patients placed in the same conditions as to age, sex, &c., we render ourselves liable to fall on the medicine which is not indicated. This is precisely what, it seems, has accidentally happened to M. Moreau.

Oxide of zinc is believed by Dr. Herpin to fail generally in epileptic patients in the vigour of their age, especially in men. Taking the whole of the cases placed in favourable conditions as regarded the number of previous attacks, and which were treated by oxide of zinc, he finds that there were twenty-six cures and five failures—all the latter being in patients between the ages of seventeen and fifty-nine years. On examining into the results of the treatment by oxide of zinc in men of between twenty and fifty years, in order that the conditions of sex and age might be the same as in M. Moreau's patients, Dr. Herpin finds six patients who were almost all in the most favourable conditions for treatment. In one, venesection appeared to have more influence than the zinc, in producing improvement. Of the remaining five cases, there were—one cure without relapse, in a patient who had had only three attacks; two cures followed by relapse—in one of these the oxide of zinc failed on the subsequent trial; one in whom improvement was produced at the age of fifteen, but in whom the same remedy failed ten years later; and lastly, one in whom the disease altogether resisted treatment, although it had been commenced five days after the first attack. Thus, while the total number of favourable cases treated by zinc are in the proportion of five to six, adults furnish only three cases out of five, and in only one of these was the cure permanent.

In adult age, it is necessary to give zinc in large doses and for a considerable time; in childhood and old age, the same result is obtained from smaller doses, and, in some cases, from almost insignificant quantities.

The preceding observations appear to Dr. Herpin to afford sufficient reason for arriving at the following conclusions:—

1. Oxide of zinc seems to be indicated as an anti-epileptic in children and old persons.
2. It often fails in persons of middle age, especially in men.
3. If it be employed in females, it must be given in large doses and for a long time.

Whatever, Dr. Herpin observes, may be the remedies employed, it is of the highest importance that the disease be treated at as early a period as possible. He is convinced that, by perseveringly treating epilepsy from its earliest manifestation, there is a certainty of cure in a large majority of cases. At present, some mistake the first symptoms of the disease; others treat it for a time by means almost always inefficacious, such as bleeding, anthelmintics, &c.; others again try useful remedies, but timidly, and without effect. A small number, chiefly hospital physicians, form a tolerably accurate notion of the choice of a medicine and of the results obtained; but they are placed in the worst conditions for acting at the most favourable moment.

Dr. Herpin promises, at a future period, to publish in the *Union Médicale* the details of some cases in the private practice of himself and others, giving both the successful and the unsuccessful cases in the proportions in which they have been met with.

